# Effects of Environmental and Pathological Hypoxia on Male Fertility

**DOI:** 10.3389/fcell.2021.725933

**Published:** 2021-09-13

**Authors:** Zhibin Li, Sumin Wang, Chunli Gong, Yiyang Hu, Jiao Liu, Wei Wang, Yang Chen, Qiushi Liao, Bing He, Yu Huang, Qiang Luo, Yongbing Zhao, Yufeng Xiao

**Affiliations:** ^1^Department of Gastroenterology, Xinqiao Hospital, Third Military Medical University, Chongqing, China; ^2^Key Laboratory of Extreme Environmental Medicine, Ministry of Education of China, Chongqing, China; ^3^Department of Endoscope, The General Hospital of Shenyang Military Region, Liaoning, China; ^4^Department of Laboratory Medicine, General Hospital of Northern Theater Command, Shenyang, China

**Keywords:** hypoxia, infertility, sperm, spermatogenic cells, spermatogenesis, HIF-1α

## Abstract

Male infertility is a widespread health problem affecting approximately 6%–8% of the male population, and hypoxia may be a causative factor. In mammals, two types of hypoxia are known, including environmental and pathological hypoxia. Studies looking at the effects of hypoxia on male infertility have linked both types of hypoxia to poor sperm quality and pregnancy outcomes. Hypoxia damages testicular seminiferous tubule directly, leading to the disorder of seminiferous epithelium and shedding of spermatogenic cells. Hypoxia can also disrupt the balance between oxidative phosphorylation and glycolysis of spermatogenic cells, resulting in impaired self-renewal and differentiation of spermatogonia, and failure of meiosis. In addition, hypoxia disrupts the secretion of reproductive hormones, causing spermatogenic arrest and erectile dysfunction. The possible mechanisms involved in hypoxia on male reproductive toxicity mainly include excessive ROS mediated oxidative stress, HIF-1α mediated germ cell apoptosis and proliferation inhibition, systematic inflammation and epigenetic changes. In this review, we discuss the correlations between hypoxia and male infertility based on epidemiological, clinical and animal studies and enumerate the hypoxic factors causing male infertility in detail. Demonstration of the causal association between hypoxia and male infertility will provide more options for the treatment of male infertility

## Introduction

The clinical definition of infertility is described as the failure of couples to conceive after more than 1 year of regular unprotected intercourse (Zegers-Hochschild et al., 2009). According to the World Health Organization statistics, infertility has become a global public health issue, affecting approximately 12%–15% of couples worldwide (Practice Committee of the American Society for Reproductive Medicine, 2015). Male factors are responsible for 50% of the cases of infertility, among which 20%–30% occur solely due to male factors, and 20%–30% are due to factors affecting both partners (Tournaye et al., 2017). Data from a global burden of disease (GBD survey suggested that the prevalence of male infertility increased by 0.291% per year from 1990 to 2017 globally (Sun et al., 2019), and may approach the 50% limit. Currently, male infertility is not just a quality-of-life problem, but a political theme with considerable social distress, and it imposes a substantial financial burden on couples and health-care systems (Agarwal et al., 2021).

The causes of male infertility are wide-ranging, including obesity, psychological stress and environmental pollutants, and hypoxia is one of the predominant reasons (Gat et al., 2006, 2005). Hypoxia is defined as a transient or sustained condition of decreased arterial oxygen partial pressure resulting in tissue oxygen deficiency, which is characterized by a decrease in arterial oxygen partial pressure and oxygen content (Reyes et al., 2012). In humans, the arterial oxygen partial pressure (PaO_2_) is 12–13.3Kpa (90–100 mmHg) and the arterial oxygen saturation (SaO_2_) is 92–96% during normal conditions at sea level. Hypoxia occurs when SaO_2_ drops below 90% (Bomhard and Gelbke, 2013). In mammals, two types of hypoxia are known, including environmental hypoxia and pathological hypoxia. The former mainly refers to low partial pressure of inhaled oxygen caused by high altitude, while the latter is impaired testicular oxygen delivery or utilization caused by pathological factors, including varicocele (Jensen et al., 2017), chronic lung disease (Semple et al., 1983), sleep apnea (Mesarwi et al., 2019) and sickle cell disease (Torres et al., 2014). Both environmental and pathological hypoxia have been shown to negatively affect male fertility in animals and humans, which can lead to a reduced sperm count, low sperm motility and abnormal sperm morphology on sperm output (Ata-Abadi et al., 2020; Wang J. et al., 2020). However, the adverse consequences of hypoxia on male fertility have been established in some studies, but it is difficult to make a firm conclusion without enough evidence. In this review, we summarize the effects of hypoxia on various aspects of fertility based on basic and clinical evidence in great detail, and discuss the potential mechanisms. Finally, we enumerate the environmental and pathological hypoxic factors causing male infertility. A correct understanding of the relationship between hypoxia and male infertility will provide more ideas about the etiological diagnosis of male infertility and more options for its treatment.

## Overview of Hypoxia

Oxygen began to accumulate in the atmosphere approximately 2.5 billion years ago and reached its present level (∼21%) about 350 million years ago (Bekker et al., 2004). The crucial function of oxygen is to act as a terminal electron acceptor to participate in aerobic respiration, which converts the chemical energy in cells into the active chemical energy in ATP through oxidative phosphorylation. Energy produced by aerobic respiration is sufficient to support physicochemical reactions in living cells and is incapable being sustained by glycolysis alone under hypoxic conditions (Semenza, 2012).

**FIGURE 1:**
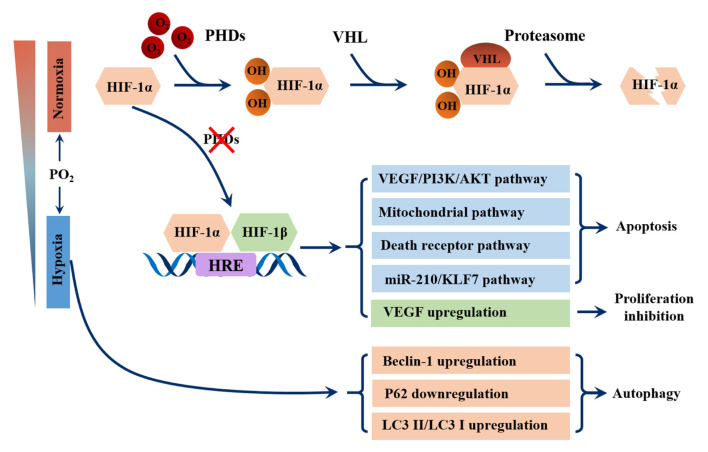
Schematic overview of the pathways involved in germ cell damage caused by HIF-1α. In the presence of oxygen, prolyl hydroxylases (PHDs) hydroxylate the HIF-1α, which generates a binding site for the von Hippel Lindau (VHL) protein, a component of the ubiquitin ligase complex, leading to proteasomal degradation of HIF-α. During hypoxia, the enzymatic activity of PHDs is inhibited, leading to stabilization of HIF-1α subunits. After translocation to the nucleus, they complex with their β subunit and bind to hypoxia responsive elements (HREs). The genes activated by HIF perform the following functions in germ cells: apoptosis, which is caused by VEGF/PI3K/AKT signaling pathway, mitochondrial pathway, death receptor pathway and miR210/KLF7 pathway; Proliferation inhibition, which is caused by activating VEGF transcription. Hypoxia can also upregulate the expression of Beclin-1 and LC3II/LC3I, and downregulate the expression of P62 to promote germ cell autophagy.

The evolution of the respiratory system and cardiovascular system allowed atmospheric oxygen to be transported to tissues directly through the bloodstream in more complex metazoans, such as *Homo sapiens*. When environmental oxygen content is low or respiratory or cardiovascular systems are impaired, hypoxia occurs (MacIntyre, 2014). Most mammals show little tolerance to hypoxia and their response involves the activation of regulatory mechanisms at systemic, tissue and cellular levels. The key factor in oxygen adaptation is hypoxia inducible factor (HIF) (Choudhry and Harris, 2018). Structurally, HIF is a heterodimer comprised of α and β subunits, and each subunit contains basic helix-loop helix PAS domains for DNA binding (Wang et al., 1995). Under normal oxygen conditions, the proline hydroxylases (PhDs) family (also known as the Egin or HPH family) hydroxylates one or both highly conserved proline residues near NTAD, which generates a binding site for the von Hippel Lindau (pVHL) tumor suppressor protein, a component of the ubiquitin ligase complex, leading to ubiquitination degradation of HIF-α (Choudhry and Harris, 2018). The PHD is inactive when oxygen is not available, allowing HIF-α to stabilize and accumulate gradually, dimerizing with HIF-β (Semenza, 2012). Upon dimerization, HIF translocates to the nucleus and binds to hypoxia response elements (HREs) to play a transcriptional regulatory role in target genes (Figure 1) (Majmundar et al., 2010).

HIF-1α mRNA is expressed equally in all male reproductive systems, including the testis, all segments of the epididymis, ductus deferens, accessory sex glands, and penis (Powell et al., 2002). In the testes of rats, immunoblot and immunohistochemical analysis revealed that HIF-1α protein was expressed in almost every epididymal epithelium and was significantly higher in hypoxic testes than in normoxic testes (Liang et al., 2015; Zhang et al., 2016). In humans, the expression of HIF-1α protein was 7 times higher in patients with varicocele (testicular hypoxia) than in normal volunteers (Lee et al., 2006). Widespread expression of HIF-1α is the basis of hypoxic adaptation in the male reproductive system.

## Reproductive Consequences of Hypoxia

### Reduced Sperm Quality

The sperm quality outcome measures are the sperm concentration, motility, morphology, semen volume, viability and DNA fragmentation, which are the parameters most frequently used in clinical settings (Schisterman et al., 2020). Growing evidence has shown that hypoxia has adverse effects on sperm quality in several species, including rodents, livestock, Drosophila, fish, and humans (Wang et al., 2016; Cofre et al., 2018; Wang J. et al., 2020). In rats and mice, acute, intermittent and chronic hypoxia reduced the total sperm concentration and sperm motility while increasing the rates of sperm DNA fragmentation and abnormal sperm morphology (Vargas et al., 2011; Torres et al., 2014; Bai et al., 2018; Wang J. et al., 2020; Ma et al., 2021). Hypoxia in rams led to a lower sperm count, sperm progressive motility and viability than in normoxic rams (Cofre et al., 2018). In humans, male soldiers exposed to hypoxia at high altitude (5380 m) for 12 months showed a poor sperm concentration, sperm motility, sperm density, and survival rate compared with those at 1 month before exposure (He et al., 2015). Similar observations were made among mountain trekkers, expeditions and specimens (Donayre et al., 1968; Okumura et al., 2003; Verratti et al., 2008). Furthermore, hypoxia mediated transgenerational impairments in sperm quality. Studies from hypoxic fish proved that exposing the Fo generation to hypoxia led to a decrease in sperm count and motility in the F1 and F2 generations that had never been previously exposed to hypoxia (Wang et al., 2016; Lai et al., 2018).

It is worth noting that patients or animals with pathological hypoxia such as sickle cell disease (Friedman et al., 1974; Nahoum et al., 1980; Osegbe et al., 1981; Agbaraji et al., 1988; Modebe and Ezeh, 1995; Berthaut et al., 2008), thalassemia (De Sanctis et al., 2008; Safarinejad, 2008a), lung diseases (Charpin et al., 1985) and obstructive sleep apnea hypopnea syndrome (Torres et al., 2014) also showed decreased sperm parameters of sperm output. Taking sickle cell disease as an example, D N Osegbe et al. measured different semen parameters including sperm motility, density, morphology, semen, viscosity, volume, pH and liquefaction time produced by 40 male patients aged 20.56 years with sick cell disease, and found that the semen parameters of all sickle cell subjects did not meet the minimum requirements for fertility (Osegbe et al., 1981). Subfertility seems to be a great problem among these patients with sickle cell disease, because they have rarely fathered children (Akinla, 1972).

### Impaired Spermatogenesis

Successful fertility requires a large amount of normal sperm. In order to meet the minimum fertility standard, the sperm concentration is greater than 15 million per ml, of which at least 40% should be motility and at least 4% with a normal morphology according to the new criteria for the laboratory examination of human sperm parameters of WHO [World Health Organization (WHO), 2010]. The generation of sperm is depended on a highly dynamic cellular differentiation process in the seminiferous tubules of the testis, called spermatogenesis (Griswold, 2016). This process begins with the self-renewal and differentiation of spermatogonial stem cells (SSCs) (Yeh et al., 2011). Self-renewal and differentiation of spermatogonial stem cells must be able to self-renew to maintain stem cell populations and undergo differentiation to form sperm, and this relationship between proliferation and differentiation rates is a parameter highly influenced by oxygen availability (Yeh et al., 2011). It is well established that cells mainly undergo oxidative phosphorylation to produce ATP in the presence of sufficient oxygen supply, while in the absence of oxygen, ATP is produced by glycolysis. However, stem cells, as well as SSCs, rely more on glycolysis for ATP production to avoid DNA damage caused by excessive ROS produced by oxidative phosphorylation, but oxidative phosphorylation and oxidative phosphorylation are also essential, since ROS are also required for SSC self-renewal (Morimoto et al., 2013; Ryall et al., 2015). This is a bioenergenic balance between glycolysis and oxidative phosphorylation, which can be disrupted in the absence of oxygen. Recent research has found that the inhibition of mitochondrial respiration and glycolysis in undifferentiated spermatogonia cells results in decreased spermatogonial colony size, and reduced expression of SSC marker genes, such as Plzf, Id4, Gfrα1, Etv5, and Sall4, suggesting that hypoxia may affect spermatogonia differentiation (Chen et al., 2020).

Following self-renewal and differentiation, SSCs transform into primary spermatocytes, which undergo two meiotic divisions to reduce the chromosome number from diploid to haploid and diversify the genetic material to form round spermatids (Lloyd and Bomblies, 2016); Considering that the seminiferous tube is thicker in diameter and lacks a vascular supply, oxygen reaches the lumen only by diffusion. Due to the limited diffusion distance and the high oxygen consumption of spermatogenesis, luminal PO_2_ is likely to be very low (Wenger and Katschinski, 2005). Therefore, spermatocytes appear to have less access to oxygen than SSCs (Cross and Silver, 1962; Wenger and Katschinski, 2005). Although few studies have focused on oxygen and energy metabolism in spermatocyte meiosis, it is undeniable that hypoxia is bound to increase the energy burden of meiosis, since meiosis is an extremely energy-intensive process. Thus it is not surprising that the apoptosis rate of spermatocytes is much higher than that of other germ cells under most hypoxic conditions (Liao et al., 2010).

Hypoxia affects not only germ cells but also the seminiferous tubule. Findings from hypoxic rats confirmed that exposing rats to hypoxic conditions arrested spermatogenic development, misshaped the arrays of spermatids (Bai et al., 2018), atrophied and thinned the seminiferous tubule lumen (Wang J. et al., 2020), decreased the cellularity of the seminiferous epithelium (Cikutovic et al., 2009; Liao et al., 2010), and disturbed the stages of the seminiferous epithelium (Gasco et al., 2003; Gonzales et al., 2004). Similar phenotypes pertaining to other animals such as mice (Kastelic et al., 2019) and rhesus monkeys (Saxena, 1995), have also been reported. In addition, Sertoli cells, the only type of somatic cells in the seminiferous epithelium of the testis that are essential for the maintenance of cell junctions, nutrient supply, and germ cells mitosis and meiosis (Ni et al., 2019), are also affected by hypoxia. A study using a hypobaric chamber simulating hypoxic condition at an altitude of 5000 m showed that 3 weeks of persistent hypoxic exposure resulted in a decrease in the number of Sertoli cells in rats (Bai et al., 2018). The number of Sertoli cells were also decreased under acute hypobaric hypoxia conditions (Shevantaeva and Kosyuga, 2006).

### Reproductive Hormone Disorders

As part of the body’s adaptation to hypoxia, the hypothalamic–adrenal–adrenal (HPA) axis regulates the functions of the hypothalamic–adrenal–gonadal (HPG) axis, which is necessary to ensure successful reproduction of males. The HPG axis influences the function of the reproductive system through the endocrine pathway, originating from the secretion of gonadotropin-releasing hormone (GnRH) in the hypothalamus. GnRH stimulates the pituitary gland to synthesize and secrete follicle-stimulating hormone (FSH) and luteinizing hormone (LH) (Neto et al., 2016). In turn, FSH and LH act on the testes to promote androgen (main testosterone) synthesis. GnRH, FSH, LH and testosterone are the major reproductive hormones that affect male reproductive functions.

The functions of FSH, LH and testosterone are mediated by their specific receptors, FSH receptor (FSHR), LH receptor (LHR), and androgen receptor (AR) respectively (Oduwole et al., 2018). FSH receptor is mainly expressed on Sertoli cells, which affects the maturation, proliferation, and function of Sertoli cells (Griswold, 1998; Abel et al., 2008). Mutations of FSH or FSHR have been associated with a decreased Sertoli cell number and sperm count, but have no effect on fertility (Tapanainen et al., 1997). LH receptor is expressed on Leydig cells to stimulate testosterone production (Narayan, 2015). The functions of testosterone are mediated by the androgen receptor (AR). Androgen receptor is generally expressed on Sertoli cells, Leydig cells and arteriole smooth muscle in the testis, but not in germ cells (Sar et al., 1990; Bremner et al., 1994; Wang et al., 2009; Smith and Walker, 2014). Findings from cell specific AR ablation or overexpression models showed that testosterone is crucial for spermatogonia number maintenance, blood-testis barrier integrity, completion of meiosis and the adhesion of spermatids and spermiation (Wang et al., 2009; Smith and Walker, 2014; O’Hara and Smith, 2015).

Through the years, although researches on hypoxia and reproductive hormones has made great progress, many contradictory reports still exist in both hypoxic animals and humans. Most studies have suggested that both environmental and pathological hypoxia decrease the levels of FSH and LH in the blood circulation (Dada and Nduka, 1980; el-Hazmi et al., 1992; Farias et al., 2008), while few studies have shown no effect or increased FSH or LH levels (He et al., 2015; Verratti et al., 2016). In addition, studies on the effects of hypoxia on testosterone secretion were also widely divergent. Several studies in humans and animals suggested that hypoxia stimulates testosterone production (Boksa and Zhang, 2008; Ma et al., 2018; Cho et al., 2019), while others suggested the opposite (Wang et al., 2017, 2019; Bai et al., 2018; Raff et al., 2018; Kim and Cho, 2019). Interestingly, similar findings were observed in cell models. One study in mouse Leydig cell line TM3 cells in a hypoxic incubator chamber showed increased testosterone release, and these effects were mediated by increased vascular endothelial growth factor (VEGF) production (Hwang et al., 2007); Another study in which rat primary Leydig cells or TM3 cells were exposed to hypoxia (1% O_2_) indicated a negative regulation of testosterone synthesis under hypoxia. This decline may be related to HIF-1α mediated transcriptional suppression of steroidogenic acute regulatory protein (Star), a rate-limiting enzyme for testosterone synthesis (Wang et al., 2018).

Actually, most of these conflicting reports are to be expected since testosterone is both a hypoventilatory and an erythropoietic hormone (Molinari, 1982; Guo et al., 2014; Marques et al., 2020). In the early stage of hypoxia (or acute hypoxia exposure), an increased in serum testosterone prevents respiratory alkalosis caused by exaggerated respiratory response of the organism (Gonzales, 2013). An increase in serum testosterone may also enhance erythropoiesis, supporting acclimatization (Gonzales, 2013). With the prolongation of hypoxia, ROS gradually accumulates in testicular cells leading to the damage of Leydig cells, which causes the decrease of testosterone. Thus it is not surprising that Madrid et al. observed an increased in testosterone during the first 24 h followed by a decrease on the 5th day in normobaric hypoxic murine model (Madrid et al., 2013).

### Erectile Dysfunction

Erectile dysfunction is a common male sexual dysfunction that is defined as the inability of the penis to attain or maintain a sufficient erection to achieve satisfactory sexual intercourse (Hatzimouratidis et al., 2010). Erectile dysfunction affects physical and psychosocial health, and has been identified as a common medical disorder over the past 20 years (Rathore et al., 2019). Causes of erectile dysfunction can be divided into 2 types: psychogenic disorders and an organic etiology. Current studies have suggested that psychogenic disorders contribute to only 20% of patients, and more than 80% of sufferers have an organic etiology (Yafi et al., 2016). There are two causes of organic etiology: endocrine and non-endocrine. Of the edocrinological erectile dysfunctions, testosterone plays important roles in enhancing sexual desire and erections (Shamloul and Ghanem, 2013); In terms of non-edocrinological erectile dysfunction, vasculogenic factors including arterial inflow disorders and corporeal veno-occlusion are the most common (Yafi et al., 2016). Notably, hypoxia can affect both types of ED mentioned above simultaneously. Hypoxia can lead to a disturbance (mostly reduction) of testosterone secretion, as described in the previous section “Reproductive hormone disorders,” which is the main cause of edocrinological erectile dysfunctions. Hypoxia from decreased corpora cavernosal oxygenation results in a decrease in prostaglandin E1 levels, which play a role in inhibiting pro-fibrotic cytokines, including transforming growth factor β1 (TGFβ1). These pro-fibrotic cytokines promote collagen deposition, replacing the smooth muscle and resulting in decreased elasticity of the penis. As the smooth muscle to collagen ratio decreases and collagen content increases, the ability of the cavernosa to compress the subtunical veins decreases, leading to corporal veno-occlusive dysfunction (Yafi et al., 2016).

Findings from several animal models of disease and clinical reports suggested that hypoxia impairs NO synthesis, which in turn decreases the functional integrity of penile smooth muscles (Moreland, 1998; Saenz de Tejada et al., 2004). A murine model of chronic intermittent hypoxia showed that 1 weeks of chronic intermittent hypoxia exposure resulted in a 55% decline in daily spontaneous erections. After 5 weeks of exposure, non-contact sexual activity was significantly suppressed, latencies for mounts and intromissions increased by 60- and 40-fold, respectively, and the sexual activity index decreased 6-fold (Soukhova-O’Hare et al., 2008). Another study performed in rats found that chronic intermittent hypoxia exposure significantly decreased the ratio of intracavernous pressure(ICP) to mean arterial blood pressure (MAP), an indicator of penile erectile response, at all levels (2.5, 5.0, and 7.5 volts) compared with normoxic rats. Furthermore, higher of apoptotic index and lower smooth muscle/collagen of corpus cavernosum were observed in hypoxic rats than normoxic (Moreland, 1998; Zhu et al., 2015). In humans, a study of hypoxia associated with idiopathic pulmonary fibrosis showed that the severity of hypoxia was closely associated with degree of testosterone suppression, which led to erectile dysfunction (Semple P. D. et al., 1984).

## Potential Mechanisms by Which Hypoxia Causes Male Infertility

### Excessive ROS Mediates Oxidative Stress

Reactive oxygen species (ROS), including superoxide anions (O2•), hydrogen peroxide (H_2_O_2_), peroxyl (ROO•), and hydroxyl (OH•) radicals, participate in almost all cell physiological processes as signaling molecules (Boveris and Chance, 1973; Du Plessis et al., 2015). In the testis, the physiological level of ROS is beneficial for SSCs self-renewal, germ cell proliferation, maturation and sperm capacitation, acrosome reaction, hyperactivation, and the fusion of spermatozoa with the oocyte (Ford, 2004; Agarwal et al., 2012; Morimoto et al., 2013; Bejarano et al., 2014; Aitken, 2017). However, excessive levels of ROS can promote the cell reductive-oxidative balance to an oxidative state, leading to oxidative stress, and thereby damaging the physiological functions of proteins, lipids and DNA.

**FIGURE 2:**
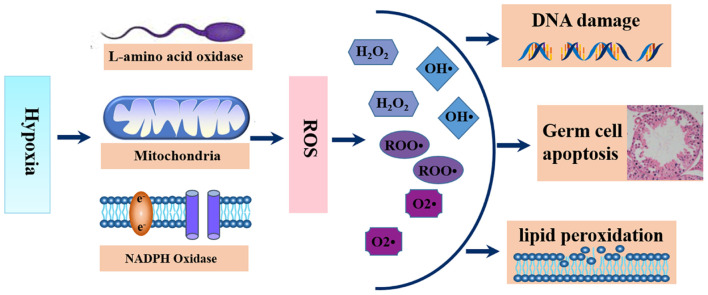
Schematic diagram of the generations and functions of ROS. Hypoxic exposure stimulates the generation of excess ROS from sperm mitochondria, cytosolic L-amino acid oxidases, and plasma membrane nicotinamide adenine dinucleotide phosphate oxidases, leading to sperm lipid peroxidation, sperm DNA damage and germ cell apoptosis.

Although conventional wisdom holds that exposure of cells to excess oxygen leads to the generation of ROS, studies have shown that exposure of cells to hypoxic conditions also leads to increased excessive ROS generation (Kim et al., 2006; Yadav et al., 2019; Zhao et al., 2020). As early as 1943, John Macleod discovered that increased production of H_2_O_2_ led to a decrease in sperm motility, this was a breakthrough that opened a pathway for research on the role of ROS in sperm function. In sperm, the sources of ROS are mainly the sperm mitochondria, cytosolic L-amino acid oxidases, and plasma membrane nicotinamide adenine dinucleotide phosphate oxidases (Aitken, 2017) (Figure 2). Findings from past studies have linked excessive ROS to poor sperm quality and male infertility (Ford, 2004; Tremellen, 2008), and up to 30%–80% of the pathology of infertility is attributed to ROS-mediated sperm damage (Aitken and Fisher, 1994; Agarwal et al., 2006, 2019). Reactive oxygen species causes infertility in two principal ways. First, ROS causes membrane lipid peroxidation, that disturbs its fluidity, resulting in damage to the sperm membrane and thus affecting sperm motility and its ability to fuse with the vitelline membrane of oocytes due to the resulting damage to the sperm membrane. Second, ROS acts directly on sperm DNA, causing DNA double or single-strand breaks, which weakens the paternal genomic contribution to the embryo (Tremellen, 2008). Data from numerous studies have highlighted that ROS had significant negative effects on spermatogenesis (Liu B. et al., 2019; Sharma et al., 2019), steroidogenesis (Chen et al., 2010; Tai and Ascoli, 2011), and epididymal sperm maturation (Arenas-Rios et al., 2016; Schneider et al., 2020). Furthermore, ROS can also mediate germ cell apoptosis by activating mitochondrial and death receptor apoptotic pathways, which may be the main cause of sperm count reductions (Ghosh and Mukherjee, 2018; Liu T. et al., 2019) (Figure 2).

### HIF-1α Mediates Germ Cell Apoptosis and Proliferation Inhibition

Programmed cell death, which we call apoptosis, is a critical biological process for regulating both the size and the quality of male germ cells and controlling the sperm output. In fact, only 25% of germ cells achieve maturity during spermatogenesis, and more than 75% of the sperm yield were lost through apoptosis in any phase of spermatogenesis (Aitken et al., 2011). Under physiological conditions, apoptosis plays an important role in the elimination of damaged germ cells, avoiding the passage of defects to future generations (Shaha et al., 2010). However, dysregulated apoptosis of germ cells was indicated in the etiology of male infertility, since increased apoptosis of cells has been observed in the testes of infertile men or animal models (Lin et al., 1997; Fouchecourt et al., 2016; Liu P. et al., 2018; Mu et al., 2018).

Previous studies have shown that hypoxia is a powerful initiator of apoptosis (Guven et al., 2014; Zhang et al., 2016), however, the mechanism by which hypoxia induces apoptosis in germ cells has yet to be defined. HIF-1α may be a key factor in mediating apoptosis. In rat models, silencing the HIF-1α gene in varicocele testes using the CRISPR/Cas9 gene editing technique significantly reduced the rate of apoptosis of spermatogenic cells and improved spermatogenic function by downregulating the VEGF/PI3K/AKT signaling pathway (Wang D. et al., 2020). Another study revealed that silencing HIF-1α significantly downregulated the expression of Bax and cleaved caspase-3 in the testes of varicocele rats (Zhao et al., 2019). In cell models, HIF-1α induced GC-2 cell apoptosis by activating the mitochondrial pathway and death receptor pathway under hypoxic conditions (Yin et al., 2018a). In addition, miR-210, a robust target of HIF-1α, also plays a crucial role in the apoptosis of germ cells (Lv et al., 2019). A study using a hypoxia workstation found that hypoxia induced miR-210 expression triggers apoptosis of mouse spermatocyte GC-2 cells by targeting Kruppel-like factor 7(KLF7), a transcription factor involved in cell proliferation (Gupta et al., 2020) (Figure 1).

Autophagy has been shown to play an important role in testicular damage under hypoxic conditions. In rats, varicocele testes cells showed increased expression of autophagy marker Beclin 1 and microtubule associated protein 1 light chain 3α (LC3) II/LC3I (Zhu et al., 2017); Exposure of GC-2 cells to hypoxia conditions reduced P62 protein expression and increased the expression of LC3 II and Beclin-1 (Yin et al., 2018b; Zhou et al., 2018). In varicocele rats, short-term hypoxia exposure promoted autophagy to stimulate testosterone secretion by degrading intracellular lipid droplets/total cholesterol. The change could be abolished by blocking autophagy (Ma et al., 2018) (Figure 1).

Notably, as a downstream target gene of HIF-1α, VEGF is upregulated under hypoxia, which is essential for endothelial growth and permeability (Apte et al., 2019). In addition to the well-known effects of VEGF, Korpelainen et al. observed that the VEGF transgene has non-endothelial target cells in the testis, leading to spermatogenic arrest and increased capillary density, which may regulate male fertility (Korpelainen et al., 1998). Another study reported that the VEGF upregulation in the testis under hypoxia suppresses the spermatogenesis by inhibiting germ cell proliferation, leading to aspermatogenesis and infertility (Nalbandian et al., 2003) (Figure 1).

### Systematic Inflammation

Findings from several studies have linked increased levels of proinflammatory cytokines to spermatogenesis disorder, poor sperm quality and infertility (Zhao et al., 2017; Beigi Harchegani et al., 2020; Cao et al., 2020). Numerous studies have shown that hypoxia is a powerful cause of inflammation. In humans, exposure to intermittent hypoxia or acute hypoxia increased the serum levels of inflammatory markers, including interleukin 1 receptor antagonist (IL-1ra), interleukin 6 (IL-6) and C-reactive protein (CRP) (Hartmann et al., 2000; Gangwar et al., 2019). Other studies on obstructive sleep apnea syndrome suggested that patients with obstructive sleep apnea syndrome showed high serum levels of tumor necrosis factor-α (TNF-α), IL-6, CRP, and spontaneous production of IL-6 by monocytes compared with obese control subjects (Vgontzas et al., 2000; Yokoe et al., 2003). Hypoxia has also been shown to be positively correlated with these proinflammatory markers in animal studies (Ren and Hu, 2017; Alshanwani et al., 2020).

**FIGURE 3:**
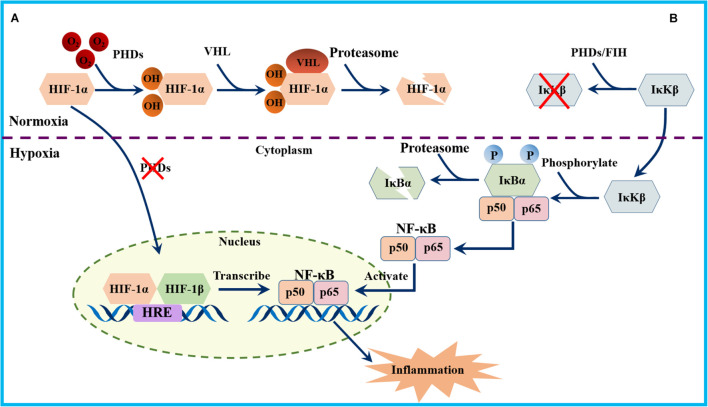
Schematic diagram of the NF-kB activation pathways. **(A)** Under hypoxia, HIF-1α is stabilized and translocate to the nucleus, dimerizing with the β subunit and binds to HREs in the promoters of NF-κB to initiate its transcription. **(B)** Under normoxia, PHDs and factor inhibiting hypoxia-inducible factor (FIH) prevent the activation of IκBβ. During hypoxia, IκKβ phosphorylate the NF-κB inhibitor IκBα, which results in its ubiquitylation and subsequent proteasomal degradation. Dissociation of IκBα allows the nuclear translocation of the NF-κB heterodimer and the transcription of its target genes.

Hypoxic exposure was suggested to promote the activity of numerous transcription factors, including nuclear factor-κB (NF-κB), a known target gene of HIF-1α (Figure 3). NF-κB is a heterodimer consisting of p50 and p65 subunits, which acts as a central transcriptional regulator of the immune response and immune cell function (Rius et al., 2008). Activation of NF-κB requires phosphorylation-induced proteasomal degradation of inhibitory IκB proteins, which is mediated by IκB kinases (IκKs) (Rius et al., 2008). Two IκKs are known, IκKα and IκKβ, in which IκKβ plays a major role in phosphorylation of IκB inhibitors. Interestingly, the evidence showed that NF-κB was activated in hypoxia through PHD-dependent hydroxylation of IκK-β (Cummins et al., 2006) (Figure 3). Furthermore, findings in animals and humans found that hypoxia -induced inflammation is characterized by elevated levels of NF-κB and the proinflammatory biomarkers IL-6, IL-1, and TNF-α (Mazzeo, 2005; de Gonzalo-Calvo et al., 2010; S et al., 2012).

### Epigenetic Changes

**FIGURE 4:**
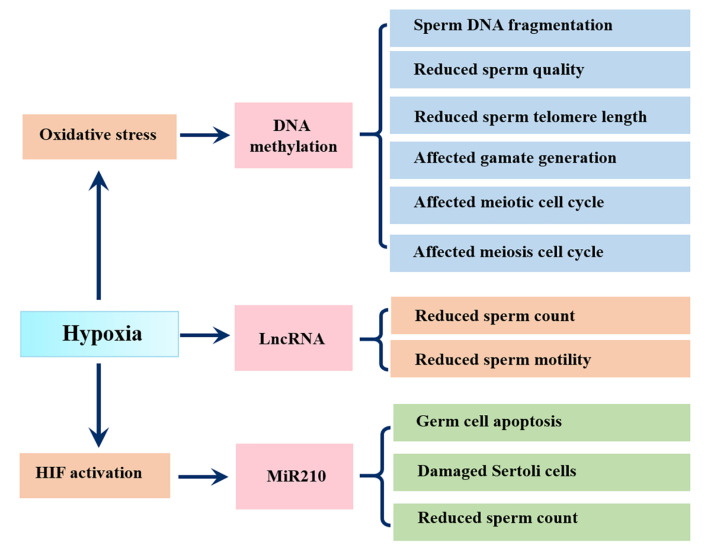
Influence of hypoxia on epigenetic changes in sperm. Hypoxia causes oxidative stress, which changes DNA methylation in sperm, resulting in sperm DNA fragmentation, reduced sperm quality, sperm telomere length and affected gamate generation and affected meiotic and meiosis cell cycle; Hypoxia related lncRNAs show negative correlations with sperm count and sperm motility; Hypoxia activates miR210, leading to germ cell apoptosis, Sertoli cell damage and reduced sperm count.

Epigenetic modifications, including histone modifications, DNA/RNA methylation and non-coding RNAs, affect the phenotype by regulating gene expression without altering the coding sequence of DNA. DNA methylation is one of the most pervasive epigenetic modification types and it remains stable during spermatogenesis (Liu Y. et al., 2019). Changing in sperm DNA methylation patterns are closely related to DNA fragmentation, reduced telomere length and male infertility (Khezri et al., 2019; Santana et al., 2019). A meta analytic study showed that the proportion of aberrant sperm DNA methylation in infertile men was 9.91 times higher than that in matched fertile men (Santi et al., 2017). Aberrant sperm DNA methylation was also found in asthenospermia or other types of male infertility (Kobayashi et al., 2007). Furthermore, reduced sperm concentration, motility and abnormal morphology were related to broad DNA hypermethylation across a number of loci (Rajender et al., 2011). Oxidative stress, an important pathological consequence of hypoxia, is a known stressor of DNA methylation (Niu et al., 2015), thus people or animals suffering from hypoxia have a higher risk of aberrant sperm DNA methylation. In this regard, Bahreinian et al. demonstrated a negative correlation between methylation and DNA fragmentation. They found that infertile men with varicocele showed lower DNA methylation as well as lower sperm parameters (sperm concentration, sperm motility, percentage abnormal morphology) and higher sperm DNA fragmentation compared with fertile men (Bahreinian et al., 2015). Interestingly, most of the differentially methylated CpG sites were hypomethylated in the varicocele group, and these regions show associations with male reproductive pathways such as semen quality, gamete generation, and meiotic and meiosis cell cycle based on gene ontology analysis (Santana et al., 2020) (Figure 4).

Notably, more powerful evidence is reflected in animal models. Wang et al. found that hypoxic treatment of F0 generation fish resulted in sperm DNA hypermethylation in the F0 and F2 generation, which was responsible for the aberrant sperm motility (Wang et al., 2016). Specifically, the exonic region of forkheadbox P2 (FOXP2), a conserved transcription factor involved in germ-cell development and spermatogenesis, was hypomethylated after hypoxic exposure (Figure 4).

Hypoxia can also affect male reproduction by regulating non-coding RNAs expression, including miR210. Mir210, also known as hypoxamiR, is a robust target of HIF and plays instrumental roles in hypoxic cell metabolism, survival, redox balance and angiogenesis (Cicchillitti et al., 2012). A study using hypoxic GC-2 cells showed that hypoxia increased miR-210 expression triggered apoptosis of GC-2 cell via activation of the apoptosis signaling pathway (Lv et al., 2019). Another study found that miR-210 was negatively correlated with the sperm count and seminal inhibin-B expression and may be an invasive biomarker of Sertoli cell damage in varicocoele (Ma et al., 2021). In addition, findings from 25 infertile patients with varicocele and 14 fertile men found that hypoxia related lncRNAs, including MLLT4-AS1 and MIR210HG, showed significantly negative correlations with sperm count and sperm motility (Ata-Abadi et al., 2020) (Figure 4).

## Hypoxic Factors Causing Male Infertility

### Environmental Factors

The Earth’s surface is surrounded by a layer of air approximately 200 km thick, called the atmosphere. The atmosphere is a mixture of various gases, of which O_2_ accounts for 21%, CO_2_ accounts for 0.027%, and N_2_ accounts for 78%, and these proportions remain balanced regardless of altitude. Dalton’s law establishes that in any given combination of gases, the total pressure is equal to the sum of the partial pressures of the gases in the mixture, so the partial pressure of oxygen(PO_2_) depends largely on the atmospheric pressure. At sea level, atmospheric pressure is about 100 Kpa. According to the Dalton’s Law, the PO_2_ can be calculated as follows:


$${\mathrm{AtmPO}}_{2}=0.21^{*}100Kpa=21Kpa$$

**FIGURE 5:**
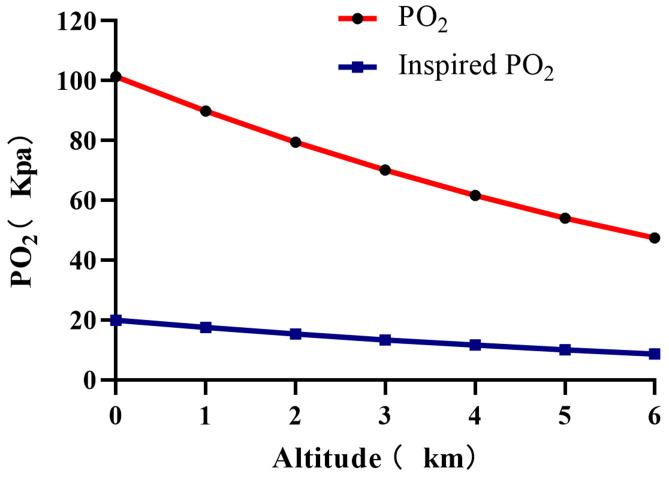
Plots of oxygen partial pressure(PO2) and inspired oxygen pressure(PiO2) at different altitudes.

Within the atmosphere, atmospheric pressure decreases with altitude, as does the PO_2_ (Figure 5). As the altitude increases, the partial pressure of inhaled oxygen decreases and hence the driving pressure of pulmonary gas exchange. Since atmospheric pressure is the sum of the partial pressure of the constituent gases, oxygen and nitrogen, and the partial pressure of water vapor (6.3 kpa at 37°C). Thus the partial pressure of inspired oxygen(PiO_2_) at sea level can be calculated as follows:


$${\mathrm{PiO}}_{2}=0.21^{*}\left(100Kpa-6.3\mathrm{Kpa}\right)=19.6\mathrm{Kpa}.$$

The partial pressure of inspired oxygen is reduced to about 13.3 kpa at 3000 m altitude, at this inspired oxygen pressure, the alveolar oxygen pressure is about 8 kPa (Peacock, 1998). At an altitude of 5500 m, the PO_2_ and PiO_2_ directly drop to 50% of the sea level, and to 30% at 8900 m (Peacock, 1998; Grocott et al., 2009) PaO_2_ dropped from 100 mmHg to 50 mmHg at 5300 m and to 24.6 mmHg at 8400 m (Grocott et al., 2009). SaO_2_ showed a decreased from 98 to 88% compared to the sea level (Bian et al., 2013).

As early as 1945, Dr. Monge found that reproduction rates among European soldiers in the Andean highlands decreased significantly, which led him to propose that hypoxia reduces fertility (Monge, 1945). In a follow-up study, He et al. found that male soldiers exposure to hypoxia at high altitude(5380 m) for 6 months causes a significantly reduction in total sperm count, sperm density, motility, survival rate and a significant prolongation of liquefaction time in young male soldiers. 12 months after hypoxic exposure, total sperm count and sperm density increased, whereas sperm motility, survival rate, and the liquefaction time further decreased. Sperm velocities, progression ratios, and lateral head displacements were also decreased (He et al., 2015). Another study of the mountain trekkers showed that 26 days of exposure at an altitude of 2000 m-5600 m resulted in lower sperm counts. Sperm motility showed no reduction immediately after returning to sea level, but decreased significantly after 1 month. Mature, normal and motile sperm in the ejaculate decreased immediately after returning to sea level and then again after 1 month (Verratti et al., 2008). Other studies involving workers, mountaineers, volunteers and border also showed significant decreases in semen quality (sperm density, motility, morphology, survival rate) after high altitude hypoxic exposure (Okumura et al., 2003; Verratti et al., 2008; He et al., 2015; Verratti et al., 2016). In addition, high altitude hypoxia also causes disorders in reproductive hormones, such as GnRH, LH, FSH, PRL and testosterone. A Finding from high altitude hypoxic male adults showed that serum levels of LH, PRL and testosterone were significantly decreased after 6 months of exposure (He et al., 2015). Another study of high altitude mountaineers revealed slightly decreased testosterone in the blood after 1 month of hypoxic exposure, which had decreased still further after 3 months (Okumura et al., 2003).

In animal models, male mice were exposed to simulated continuous or intermittent hypoxia of 4,200 m in a chamber for 33.2 days. Reproductive parameters analysis showed that there were decreased sperm count and increased teratozoospermia, sperm DNA fragmentation and the instability of DNA after hypoxic exposure (Vargas et al., 2011). Several hypoxia studies in rats exposed to high altitude have shown decreased semen parameters, reduced testis weight, degeneration of the germinal epithelium, sloughing of germ cells and Leydig cells, impairment of spermatogenesis and steroidogenesis (Gosney, 1984; Biswas et al., 1985; Gasco et al., 2003; Farias et al., 2005, 2008; Grocott et al., 2009). Similar phenotypes pertaining to other animals such as ram (Cofre et al., 2018), toad (Biswas et al., 1985) and rhesus monkeys (Saxena, 1995), have also been reported. (A summary of the studies in the last 20 years is shown in Table 1).

**TABLE 1:** Summary of the studies reporting the reproductive consequences of high altitude.

Altitude	Subjects	Reproductive consequences	References
7821 m	Human	Decreased sperm count; increased abnormally shaped sperm; decreased testosterone levels	Okumura et al., 2003
4340 m	Rat	Decreased epididymal sperm count; disorder of spermatogenesis	Gasco et al., 2003
4340 m	Rat	Reduced spermiation (stage VIII) to half and the onset of spermatogenesis (stages IX-XI) to a quarter; decreased sperm count	Gonzales et al., 2004
3400 m	Rat	Decreased epididymal spermatozoa count; reduced diameter of the seminiferous tubule and the height of the spermatogenic epithelium	Cikutovic et al., 2009
5000 m	Rat	Decrease of cellularity of seminiferous epithelium; degeneration and sloughing of seminiferous epithelial cells occasionally.	Liao et al., 2010
5300 m	Human	Decreased sperm mtDNA copy numbers; lower nuclear DNA integrity	Luo et al., 2011
5900 m	Human	Reduced sperm concentration	Pelliccione et al., 2011
4200 m	Mouse	Testicular damage	Vargas et al., 2011
5380 m	Human	Decreased sperm parameters; decreased LH, PRL and testosterone levels	He et al., 2015
3600 m	Ram	Decreased sperm concentration, progressive motility and viability	Cofre et al., 2018
3600 m	Human	Decreased sperm concentration; increased the occurrence and frequency of sperm with excessive head size, neck crimp, and tailless	Zheng et al., 2019
5500 m	Rat	Decreased sperm count and motility; increased sperm deformity rate; decreased testosterone levels	Liu et al., 2020

### Pathological Factors

#### Varicocele

Testicular varicocele is a kind of vascular disease that refers to the abnormal expansion, elongation and tortuousness of the variegated venous plexus within the spermatic cord. During the occurrence of varicocele, the blood flow system of the testicular spermatic vein is damaged, leading to venous blood stasis, which increases the hydrostatic pressure in the testis and exceeds the microcirculation pressure of the testicular artery, resulting in testicular hypoxia (Gat et al., 2010a). Approximately 15% of men worldwide have varicocele (Thomason and Fariss, 1979), and 19%–41% of them show primary infertility and 45%–81% show secondary infertility (Jarow et al., 1996). More than 50 years of studies have found that varicocele has a negative impact on sperm parameters, semen function, reproductive endocrine factors and testicular function in men.

Hypoxia is one of the most pathological processes of varicocele (Gat et al., 2010b). In patients with varicocele, testicular venous blood flow is blocked, resulting in a local hypoxia. This process is accompanied by the accumulation of HIF-1α (Zhang et al., 2016; Goren et al., 2017). A study involving 20 infertile men with varicocele and 20 fertile men showed that the sperm concentration, motility, morphology and sperm DNA integrity of varicocele men were significantly lower than those of fertile men, and molecular markers associated with the hypoxia pathway were significantly higher than those associated with the inflammation pathway, suggesting that hypoxia may be the main cause of infertility (Ghandehari-Alavijeh et al., 2019). Subsequent studies showed that hypoxia-related lncRNA expression is significantly elevated in semen from varicocele patients (Ata-Abadi et al., 2020). Mechanistically, oxidative and heat stress caused by hypoxia or hypoxia-ischemia and HIF-1α mediated germ cell apoptosis or sperm damage may be the main reasons (Zhang et al., 2016; Goren et al., 2017; Zhu et al., 2017; Samanta et al., 2018; Zhao et al., 2019). (A summary of the studies in the last 5 years is shown in Table 2).

**TABLE 2:** Summary of the studies reporting the reproductive consequence of varicocele.

Subjects	Reproductive consequences	References
Human	Poorer semen quality; higher serum levels of FSH; lower inhibin B; higher levels of LH	Damsgaard et al., 2016
Human	Sperm DNA damage	Ni et al., 2016
Human	Smaller testis; higher frequency of abnormal epididymis	Zhang et al., 2017
Human	A reduction in the concentration/mL and the total sperm number	Pallotti et al., 2018
Human	Lower sperm production and motility; increased percentage of abnormal sperm morphology	Oliva and Multigner, 2018
Rat	lower sperm count and survival rate; disordered seminiferous epithelium	Zhao et al., 2019
Human	Lower sperm parameters, sperm and leukocyte telomere length; higher DNA fragmentation, protamine deficiency, and lipid peroxidation	Tahamtan et al., 2019
Human	Higher sperm DNA fragmentation index	Nguyen et al., 2019
Human	Lower total and total motile sperm counts; slightly higher testosterone levels	Redmon et al., 2019
Human	Non-obstructive azoospermia	Kavoussi et al., 2019
Human	Decreased sperm quality	Santana et al., 2020
Human	Higher DNA fragmentation index; decreased all semen characteristics; an abnormality of at least one of the spermatic parameters	Jellad et al., 2021
Human	Higher sperm DNA fragmentation and apoptosis rate	Ammar et al., 2021
Human	Reduced semen parameters; increased sperm DNA fragmentation	Finelli et al., 2021
Human	Lower sperm concentration, motility and total sperm count;higher serum FSH levels, and higher seminal oxidation-reduction potential and sperm DNA fragmentation index	Tanaka et al., 2020

#### Chronic Lung Diseases

Chronic lung disease, such as chronic obstructive pulmonary disease(COPD), interstitial lung disease, asthma, emphysema, lung cancer and sleep apnea, can reduce ventilation-perfusion which may lead to clinically relevant hypoxemia. Hypoxemia can disturb the functions of the hypoventilation-pituitary-gonadal axis, resulting in sex hormone suppression and sexual dysfunction (Semple et al., 1981; Semple P. A. et al., 1984; Semple P. D. et al., 1984).

An autopsy study involving 10 men with hypoxia associated with bronchitis and emphysema lasting at least 15 years found that the total volume of Leydig cells in testis was significantly less than the volume in the matched control group. This atrophy may be a consequence of hypoxic inhibition of pituitary synthesis or the release of LH (Gosney, 1987). Another epidemiological study of 35 patients (24 males, 11 females) with primary bronchiectasis and 71 patients (54 males, 17 females) with secondary bronchiectasis showed a strong association between primary bronchiectasis and male infertility (Charpin et al., 1985). Other studies also demonstrated that testosterone deficiency is common in patients with COPD (Baillargeon et al., 2019). (A summary of the studies in the last 20 years is shown in Table 3).

**TABLE 3:** Summary of the studies reporting the reproductive consequences of chronic lung diseases.

Types of diseases	Subjects	Reproductive consequences	References
COPD	Human	Hypogonadism	Van Vliet et al., 2005
COPD	Human	Hypogonadism	Laghi et al., 2005
COPD	Human	Erectile dysfunction	Karadag et al., 2007
Pulmonary obstruction	Human	Reduced total and free testosterone levels	Svartberg et al., 2007
COPD	Human	Lower testosterone levels	Akbas et al., 2010
COPD	Human	Sexual dysfunctions	Collins et al., 2012
COPD	Human	Hypogonadism	Balasubramanian and Naing, 2012
COPD	Human	Lower testosterone levels	Atlantis et al., 2013
COPD	Human	Significant reduction in total and free testosterone levels	Karakou et al., 2013
COPD	Rat	Decreased testosterone levels	Wang et al., 2019
COPD	Human	Serum testosterone depression	Rubinsztajn et al., 2019
Pulmonary emphysema	Mesocricetus auratus	Decreased sperm quality; increased abnormal seminiferous tubules; decreased seminiferous epithelium height; decreased sertoli cells; increased unclear volume of leydig cells	Vieira et al., 2020

#### Obstructive Sleep Apnea Syndrome

Obstructive sleep apnea syndrome (OSAS) is a common clinical condition that is characterized by recurrent closure of the upper airway during sleep (Levy et al., 2015). Chronic intermittent hypoxia is a hallmark of OSAS, and is an important pathogenic factor for male infertility (Kiernan et al., 2016). One study looking at the effects of obstructive sleep apnea on male sexual function investigated 24 men referred for sleep research. Significantly reduced serum testosterone levels were documented in 15 men with obstructive sleep apnea (34.5 apneas/h) and 9 non-obstructive sleep apnea snorers(< 5 apneas/h), and this decrease was associated with lower min SaO_2_ but not with other demographic, respiratory or sleep parameters (Santamaria et al., 1988). In another study with 5 OSA patients and 5 healthy middle-aged controls, Luboshitzky et al. found that patients with OSA had significantly higher PaO_2_ < 90% values compared with the control groups. As expected, OSA patients had significantly lower testosterone values than in matched controls (Luboshitzky et al., 2005). In addition, a prospective cross-sectional analysis of 401 OSA patients showed that erectile dysfunction was found in 69% of patients with OSA. Stepwise multiple regression analysis revealed that mean SaO_2_ was independently associated with erectile dysfunction (Budweiser et al., 2009). Another study investigating the correlation between OSAS and erectile dysfunction showed that the prevalence of erectile dysfunction in patients with OSAS (19 of 32, 59.3%) was significantly higher than in the matched control group (8 of 27, 29.6%). Erectile dysfunction was significantly associated with the lowest oxygen saturation decreased but not apnea–hypopnea index (Shin et al., 2008).

In animal models, male mice were subjected to chronic intermittent hypoxia (20 s at 5% O_2_ followed by 40 s of room air, 6 h/day) in a gas controlled box with a frequency equivalent to sixty apneas per hour to simulate severe OSA. As expected, the male mice treated with OSA model experienced cyclic changes in SaO_2_ ranging from maxima of 95.4 ± 0.1% (similar to baseline values) to minima of 62.3 ± 3.5% (*P* < 0.001), After 60 days of treatment, Torres et al. found that chronic intermittent hypoxia significantly decreased progressive sperm motility, the proportion of pregnant females and the number of fetuses per mating. Testicular oxidative stress levels were also increased compared with those of normoxic controls (Torres et al., 2014). In rats, Wang et al. found that male rats treated with obstructive sleep apnea hypopnea syndrome model (20–21% O_2_ to 6–7% O_2_ for 30 s; 6–7% O_2_ to 20–21% O_2_ within 20 s; 20–21% O_2_ for 60s, 8h/day, 6 weeks) showed a decreased total sperm count and sperm motility and more structurally abnormal spermatogenic tubules (Wang J. et al., 2020). Thinned, arranged unevenly and atrophied spermatogenic tubules lumen, and the increased gap between the tubules was also observed in the obstructive sleep apnea hypopnea syndrome group (Wang J. et al., 2020). (A summary of the studies in the last 20 years is shown in Table 4).

**TABLE 4:** Summary of the studies reporting the reproductive consequence of sleep apnea.

Subjects	Reproductive consequences	References
Human	Reduced testosterone levels	Gambineri et al., 2003
Human	Decrease in erectile function	Margel et al., 2004
Human	Erection problems; decreased overall sexual satisfaction	Stannek et al., 2009
Human	Erectile dysfunction	Budweiser et al., 2009
Human	Erectile dysfunction; low testosterone levels	Andersen et al., 2010
Human	Reduced free testosterone and sexual quality	Hammoud et al., 2011
Mouse	Decreased testosterone levels	Wang et al., 2019
Mouse	Reduced progressive sperm motility; downregulate fertility rate and fetuses number	Torres et al., 2014
Rat	Reduced sperm count and sperm motility; impaired spermatogenesis	Wang J. et al., 2020
Human	Higher infertility rate	Chen et al., 2021

#### Hematological Diseases

In humans, oxygen is exchanged in the alveoli of the lungs. More than 95% of oxygen is delivered into the capillary vessels via the alveolar–capillary exchange system and binds to hemoglobin. The heart pumps oxygenated blood to the periphery, which is crucial for organs and cells to function and perform oxidative phosphorylation. Cardiovascular and hematological disorders can cause a decrease in blood oxygen carrying capacity or blood circulation capacity, resulting in hypoxia (Joseph et al., 2021).

Sickle cell disease (SCD) is one of the most common, inherited hematological diseases caused by a single amino acid substitution (GTG for GAG) in the gene encoding hemoglobin β (Papageorgiou et al., 2018). This substitution results in an abnormal oxygen-carrying protein, called sickle hemoglobin. Abnormal polymerization of sickle hemoglobin is responsible for vasoocclusion of testicular blood vessels, which affects oxygen delivery to the tissues and causes tissue hypoxia. A cross-sectional study of 34 male patients with sick cell disease showed that 8 men (24%) developed hypogonadal disease, characterized by decreased levels of testosterone, FSH and LH (Taddesse et al., 2012). Similar details have been found in the clinical laboratory (Dada and Nduka, 1980). Interestingly, a case report indicated that sickle cell disease caused repeat testicular infarction (Li et al., 2003). In addition, several studies have reported that hypogonadism and poor sperm parameters including low sperm counts, impaired motility of spermatozoa and increased abnormal sperm morphology occur frequently in male patients with sickle cell disease (Nahoum et al., 1980; Osegbe et al., 1981; Grigg, 2007; Berthaut et al., 2008; Joseph et al., 2021). Surprisingly, priapism and impotence occur frequently in patients with SCD, with an incidence up to 50% (Emond et al., 1980; Adeyoju et al., 2002; Nolan et al., 2005; Madu et al., 2014; Salonia et al., 2014; Chinegwundoh et al., 2017, 2020).

Beta-thalassemia is a hereditary blood disorder caused by reduced (β +) or absent (β0) synthesis of the β-globin chains of hemoglobin. The variation in hemoglobin results in a reduction in oxygen affinity and oxygen carrying capacity, resulting in tissue hypoxia (Machogu and Machado, 2018). A study involving 168 men aged 18 years or older with homozygous beta-thalassemia and 84 healthy age matched male volunteers showed that the incidence of hypogonadotropic hypogonadism was as high as 76% (128 men). Total sperm count, sperm motility, abnormal sperm morphology and serum LH, FSH, and T were lower in patients homozygous for beta-thalassemia than in normal controls (Safarinejad, 2008b). Furthermore, thalassemic patients had more sperm DNA damage than the controls (Perera et al., 2002; Elsedfy et al., 2018). (A summary of the studies in the last 20 years is shown in Table 5).

**TABLE 5:** Summary of the studies reporting the reproductive consequences of hematological diseases.

Types of diseases	Subjects	Reproductive consequences	References
Sickle cell disease	Human	Priapism	Adeyoju et al., 2002
Sickle cell disease	Human	Priapism or erectile dysfunction	Madu et al., 2014
Sickle cell disease	Human	Lower testicular volume; shorter penis length	Martins et al., 2015
Sickle cell disease	Human	Impaired sperm parameters	Joseph et al., 2021
Sickle cell disease	Human	Repeated testicular infarction	Li et al., 2003
Sickle cell disease	Human	Decreased semen parameters	Berthaut et al., 2008
Thalassemia	Human	Lower total sperm count, sperm motility and percent normal sperm morphology; lower serum LH, FSH and testosterone levels	Safarinejad, 2008b
Sickle cell disease	Human	Hypogonadism	Taddesse et al., 2012
Sickle cell disease	Human	Hypogonadism	Morrison et al., 2013
Thalassemia	Human	Increased sperm DNA damage; reduced sperm motility	Perera et al., 2002
Hemolytic anemia	Mouse	Reduced sperm count, sperm natural morphology, sperm motility and viability and serum testosterone concentration, increased DNA injury	Mozafari et al., 2016
Thalassemia	Human	Lower testis values; lower sperm concentrations and abnormal morphology	Chen et al., 2018
Hemolytic anemia	Mouse	Testicular tubular atrophy and edema in the interstitial tissue; decreased sperm count, diminished sperm motility and viability, diminished fertilizing potential	Anbara et al., 2018

## Concluding Remarks

In this review, the available evidence clearly indicates that hypoxia, both environmental and pathological, has deleterious effects on sperm parameters, testicular function, reproductive hormone secretion and pregnancy outcomes for males. Excessive ROS mediated oxidative stress, HIF-1α mediated germ cell apoptosis and proliferation inhibition, systematic inflammation and epigenetic changes seemed to be the central mechanisms. Given that infertility has become a global problem affecting human development, it is necessary to further study the molecular mechanism of infertility and search for molecular targets to reduce the burden of disease. Demonstration of the causal association between hypoxia and male infertility will provide more options for the treatment of male infertility.

## Author Contributions

ZL, SW, and CG wrote the manuscript. YHu, JL, and WW revised the manuscript. YC and QLi was responsible for searching the references. BH, YHua, and QLu projected and edited the manuscript. YZ and YX reviewed the manuscript. All authors read and approved the final manuscript.

## Conflict of Interest

The authors declare that the research was conducted in the absence of any commercial or financial relationships that could be construed as a potential conflict of interest.

## Publisher’s Note

All claims expressed in this article are solely those of the authors and do not necessarily represent those of their affiliated organizations, or those of the publisher, the editors and the reviewers. Any product that may be evaluated in this article, or claim that may be made by its manufacturer, is not guaranteed or endorsed by the publisher.
